# [Retracted] circ_103809 promotes breast cancer progression by regulating the PI3K/AKT signaling pathway

**DOI:** 10.3892/ol.2025.15176

**Published:** 2025-07-07

**Authors:** Xiaoli Qiu, Qinghua Wang, Huiping Song, Di Shao, Jie Xue

Oncol Lett 19: 3725–3730, 2020; DOI: 10.3892/ol.2020.11507

Following the publication of this paper, it was drawn to the Editor's attention by a concerned reader that certain of the colony formation assay data shown in Fig. 2B on p. 3727 had already appeared in a previously published article in the journal *Oncogene* that was written by different authors at different research institutes. Owing to the fact that the contentious data in the above article had already been published prior to its submission to *Oncology Letters*, the Editor has decided that this paper should be retracted from the Journal. After contacting the authors, they accepted the decision to retract the paper. The Editor apologizes to the readership for any inconvenience caused.

